# Therapeutic Use of LSD in Psychiatry: A Systematic Review of Randomized-Controlled Clinical Trials

**DOI:** 10.3389/fpsyt.2019.00943

**Published:** 2020-01-21

**Authors:** Juan José Fuentes, Francina Fonseca, Matilde Elices, Magí Farré, Marta Torrens

**Affiliations:** ^1^ Institut de Neuropsiquiatria i Addiccions, Hospital del Mar, Barcelona, Spain; ^2^ Addiction Research Group (GRAd), Neuroscience Research Program, Hospital del Mar Medical Research Institute (IMIM), Barcelona, Spain; ^3^ Psychiatry Department, Universitat Autònoma de Barcelona (UAB), Cerdanyola del Vallès, Spain; ^4^ Centro de Investigación Biomédica en Red de Salud Mental, CIBERSAM, Madrid, Spain; ^5^ Clinical Pharmacology Department, Hospital Universitari Germans Trias i Pujol (IGTP), Badalona, Spain; ^6^ Pharmacology Department, Universitat Autònoma de Barcelona (UAB), Cerdanyola del Vallès, Spain

**Keywords:** lysergic acid diethylamide (LSD), hallucinogens, therapeutic use, psychiatric disorders, addiction

## Abstract

Lysergic acid diethylamide (LSD) was studied from the 1950s to the 1970s to evaluate behavioral and personality changes, as well as remission of psychiatric symptoms in various disorders. LSD was used in the treatment of anxiety, depression, psychosomatic diseases and addiction. However, most of the studies were not performed under contemporary standards, and it has taken several decades for a resurgence of interest in LSD research and its therapeutic potential for psychiatry. The aim of this review is to identify controlled and randomized clinical trials that assess the potential use of LSD in psychiatry. PRISMA guidelines for systematic review were followed. A literature search of PubMed and Psychedelic bibliography from Multidisciplinary Association for Psychedelic Studies (MAPS) databases was performed as well as a manual search of references from evaluated studies. Only randomized-controlled clinical trials were included. Study quality was systematically calculated by using the Cochrane Collaboration Tool for assessing risk of bias. A final selection of 11 articles was made after considering inclusion and exclusion criteria. LSD was administered to 567 patients in a dose ranging from 20 to 800 mcg. Despite the design heterogeneity of clinical trials, positive results were observed, thus revealing the therapeutic potential of LSD to reduce psychiatric symptomatology, mainly in alcoholism. The vast majority of authors describe significant and positive short-term changes in patients, despite the fact that in some studies an important homogenization was observed between the LSD treatment group and control group at long-term follow-up. Multiple variables regarding LSD treatment therapeutic approach and quality of experience were revealed and related to therapeutic outcomes. LSD is revealed as a potential therapeutic agent in psychiatry; the evidence to date is strongest for the use of LSD in the treatment of alcoholism. Despite the difficulty of designing proper double blind clinical trials with this substance, new studies that conform to modern standards are necessary in order to strengthen our knowledge on its use and open new doors in the future.

## Introduction

Since its discovery in 1938 by Swiss chemist Albert Hofmann (1), lysergic acid diethylamide (lysergide, LSD) has maintained an unstable relationship with psychiatry. Hofmann synthesized LSD in an effort to develop ergot derivatives with the goal of reducing postpartum hemorrhage. Some years later, after accidentally getting into contact with a small dose, he was the first subject in history to experience its effects (2). At the end of the 1940s, there was great interest among psychiatrist in the potential use of LSD as a therapeutic agent (3), which was actually marketed by Sandoz laboratories under the brand name “Delysid” in the 1950s (4) and used in several psychiatric departments in Europe and America. Even the US Army and CIA experimented with this substance as a truth serum, and LSD was further investigated by the US Army as a potential incapacitating agent, however without success (5). After its prohibition in USA in 1967, due to an increase in popularity and its association with counter-cultural movements, it has taken several decades for a resurgence of interest in its therapeutic potential for psychiatry (6–9).

LSD is part of the pharmacological group known as “classical hallucinogens” or “psychedelics” (term coined by Osmond in 1957) (4), sharing its chemical structure with psilocybin and dimethyltryptamine (DMT) as a variant of indolamine (chemical structure similar to the neurotransmitter serotonin) (10).

The term “classical hallucinogen” is a widely accepted synonym in the literature, with a greater emphasis on the alteration of the perception that these substances cause (11), although its use has been controversial as it does not specify the effect of these agents in consciousness and the self, as indicated by recent psychological and biological studies (12–14). LSD could also be defined, from an anthropological perspective, as an “entheogen”, which implies that users experience (mainly in a religious, shamanic or spiritual context) an altered state of consciousness: “as if the eyes had been cleansed and the person could see the world as new in all respects” (15).

Classical hallucinogens are psychoactive substances that are believed to mediate their effects mainly through an agonist activity in the serotonin 2A receptor (5-HT2A) (16). Experimental studies have previously shown that the use of 5-HT2A antagonists attenuate the main effects of these substances, both in rats (17, 18) and human subjects (19–22).

Other receptors which may contribute to the effects of these agents are the serotonin 2C and 1A receptors, as well as other effects in the dopaminergic and noradrenergic system (16). Likewise, these are potent regulators of transcription factors, which could mediate a potential mechanism of action in the synaptic structure with greater persistence of their effects over time (23, 24).

LSD is one of the most potent classical hallucinogens available, with active doses between 0.5 and 2 mcg/kg (100–150 mcg per dose). Its half-life is approximately 3 h, varying between 2 and 5 h, and its psychoactive effects are prolonged over time (up to 12 h depending on the dose, tolerance, weight and age of the subject) (25, 26). Recently LSD has been used in microdoses as low as 10 mcg to enhance performance (27).

The usual mental effects of LSD are distortion of sense of time and identity, alteration in depth and time perception, visual hallucinations, sense of euphoria or certainty, distorted perception of the size and shape of objects, movements, color, sounds, touch and body image and delusions (28).

Concerning safety, the administration of classical hallucinogens carries some risks. One of them is the so-called “bad trip” or “challenging experience”, described as an acute state of anxiety, dysphoria and confusion, which can lead to unpredictable behavior in uncontrolled or unsupervised environments (29). Another possible risk is the exacerbation of psychotic disorders or the generation of prolonged psychotic reactions, which could be related to the subject's previous predisposition (30). Although no contemporary study has reported psychosis after the administration of classical hallucinogens, an adequate screening of previous psychotic episodes and the patient's vulnerability is necessary for the use of these substances (31). Another possible adverse effect is a modest increase in blood pressure and heart rate; therefore, patients with severe cardiovascular disease should be excluded from the administration of this agent. Other usual absolute contraindications are pregnancy, epilepsy or paranoid personality traits (32). The remaining adverse effects should not limit its therapeutic use (31, 33).

As a recreational drug, LSD does not entail physical dependence as withdrawal syndrome, as do most of these substances (opioids, cocaine, cannabis and methamphetamine) (34). Its frequent or long-term use can lead to tolerance, and after a single dose, emotional, physical and mental stability is quickly recovered (35, 36). Likewise, classical hallucinogens in general, and LSD in particular, exhibit very low physiological toxicity, even at very high doses, without any evidence of organic damage or neuropsychological deficits (36, 37) associated with their use. Their safety has recently led to considering LSD as one of the safest psychoactive recreational substances (38–42).

However, LSD remains one of the most stigmatized and legally restricted agents among psychoactive substances. It is still included in Schedule I of the United Nations classification of drugs, restricting its use in research and making it difficult to potentially use it as a therapeutic tool in medicine. This classification has recently been questioned by various authors (8, 43). A few decades ago, anecdotal reports of suicidal acts in recreational users were published, and intensely emphasized by the media (44, 45). These attempts are in contrast with some recent population studies, which show significant associations between the use of a single dose of classical hallucinogens and a decrease in the likelihood of psychological distress and suicide (46–48). Other recent studies also established a clear link between life-time use of classical hallucinogens and a lower probability of developing mental problems, as well as a positive association, although non-significant, regarding several variables related to mental health (49, 50). Nevertheless, the unpredictability of subject behavior makes it necessary to adequately control the environment and monitor the reaction of each individual.

Regarding its therapeutic potential, LSD was used from the 1950s to the 1970s to achieve behavioral and personality changes, as well as remission of psychiatric symptoms in various disorders (30, 51). LSD was used in the treatment of anxiety, depression, psychosomatic diseases and addiction (52). During that time, it was also observed that LSD together with suitable accompaniment during its administration, could reduce pain, anxiety and depression in patients with advanced cancer (53–55) Other studies involving larger patient samples also established its safety and promising results in patients with terminal cancer (56, 57). Studies in schizophrenic patients, however, reached less response to the same dose (58) and worse clinical outcomes (59) compared with non-schizophrenics patients, and negative effects on these patients have been described, both in LSD experience itself and later benefits (60, 61). The data indicate that the responsivity of schizophrenic patients to the administration of lysergic acid is less than that of normal subjects.

Prediction of individual responses to LSD depends on several variables, some of which were already discussed at the international LSD therapy conference in 1965 (52). LSD reaction involves a series of complex interactions between doses, “set” (thoughts, mood and expectations of the subject prior to treatment) and “setting” (the physical and interpersonal environment in which the subject undergoes treatment) (30). Three different major approaches to LSD use as a treatment were then applied to clinical research: “psycholytic therapy”, “psychedelic-chemotherapy” and “psychedelic-peak therapy” (62). In psycholytic therapy, mainly practiced in Europe, low-moderate doses (25-200 mcg) of this drug were used in more than one therapeutic session of psychodynamic orientation. In psychedelic-chemotherapy, drug use itself was emphasized at relatively high doses (200 mcg or more), with a very limited or absent psychotherapeutic approach. As for psychedelic-peak therapy (or “psychedelic therapy”), it involves administering a single and relatively high dose with the aim of triggering a mystical-type experience (“peak experience” or “ego dissolution” as synonyms). This approach should include the proper prior preparation of the patient (set) and a comfortable environment during the session (setting), as well as a discussion on it during subsequent follow-up sessions with the subject (after-care related to LSD session) (63). Mystical experiences are referred to as those in which a sense of unity with the environment is experienced achieving a vivid transcendental experience at an emotional, cognitive and ego-structural level, after a previous and personal therapeutic preparation (64). The aim is to catalyze rapid and fundamental changes in the value system and self-image of the subject (65).

Despite the foregoing, most clinical studies involving the use of LSD were published between the 1960s and 1970s, up to the strict prohibition of its use in research. Obviously, most of these studies were not performed under contemporary standards. The purpose of this systematic review is to identify controlled and randomized clinical trials that assess the potential use of LSD in psychiatry and identify variables controlled by the researcher as potentially related to therapeutic outcomes. This is with the aim of informing a discussion on the benefits and challenges of integrating contemporary classic hallucinogens research into modern clinical trial designs and providing a guide for further research involving LSD as a therapeutic agent.

## Methods

### Data Acquisition and Search Strategy

This study was conducted according to the requirements established in the Preferred Reporting Items for Systematic Review and Meta-Analysis (PRISMA) protocols (66).

Pubmed database was searched for the following terms: [“lysergic acid diethylamide” OR “LSD” OR “lysergic acid diethylamide” (MeSH Terms)] OR “lysergic acid”) AND [“therapeutics”(MeSH Terms) OR “mental disorder” (MeSH Terms) OR “therapy” OR “psychotherapy” OR “treatment”]. In addition, the Multidisciplinary Association for Psychedelic Studies (MAPS) Psychedelic Bibliography (www.maps.org) was also consulted. To ensure literature saturation, the electronic search was supplemented by a manual review of the reference lists from eligible publications. Two authors independently screened the titles and abstracts yielded by the search against the inclusion criteria. Full reports for all titles that appear to meet the inclusion criteria were obtained. Reviewers resolved disagreements by discussion. The search was limited to the time period compressed between 01-01-1950 and 05-05-2019, based on the results obtained in the reference search.

Search results were examined by two authors (JJF and FF) reading the titles and abstracts. Each potentially relevant publication found during the search was retrieved and assessed for its use in this review after inclusion and exclusion criteria were specified.

#### Data Items

Dosage, frequency and duration of the treatment, for both experimental and control interventions were extracted. Patient's characteristics (including age, gender and diagnosis) and inclusion/exclusion criteria were extracted together with country, trial design, trial size, and length of follow up. For non-pharmacological comparators, type, frequency and duration of the intervention were extracted, if appropriate.

As studies with different diagnostic groups were included, outcomes varied depending on the psychiatric condition under study. In any case, change scores from baseline or endpoint were extracted. Side effects and overall tolerability were also studied.

### Eligibility Criteria

Randomized controlled trials of LSD as a therapeutic tool for psychiatry were included. This review included only randomized controlled clinical trials involving patients with a diagnosis of mental illness. Experimental studies in healthy volunteers were excluded. Trials with no control group or not randomized, animal studies, observational studies, review papers, qualitative studies, case reports, opinion pieces or comments, letters or editorials, conference abstract, posters and books chapters were excluded. Of interest were interventions using LSD, as a stand-alone treatment or as an adjunctive treatment. Only studies comparing LSD with other interventions were included. Active and non-active comparators were included.

### Quality Assessment

The Cochrane Collaboration risk of bias assessment tool was used to determine the quality of the studies (67). This tool involves an assessment of six specific domains: 1) sequence generation, 2) allocation concealment, 3) blinding of participants, 4) personnel and outcome assessors, 5) incomplete outcome data, and 6) selective outcome reporting and other sources of bias. The tool was applied to each RCT independently by two authors. Discrepancies were resolved through discussion with a third author.

## Results

**Figure 1:**
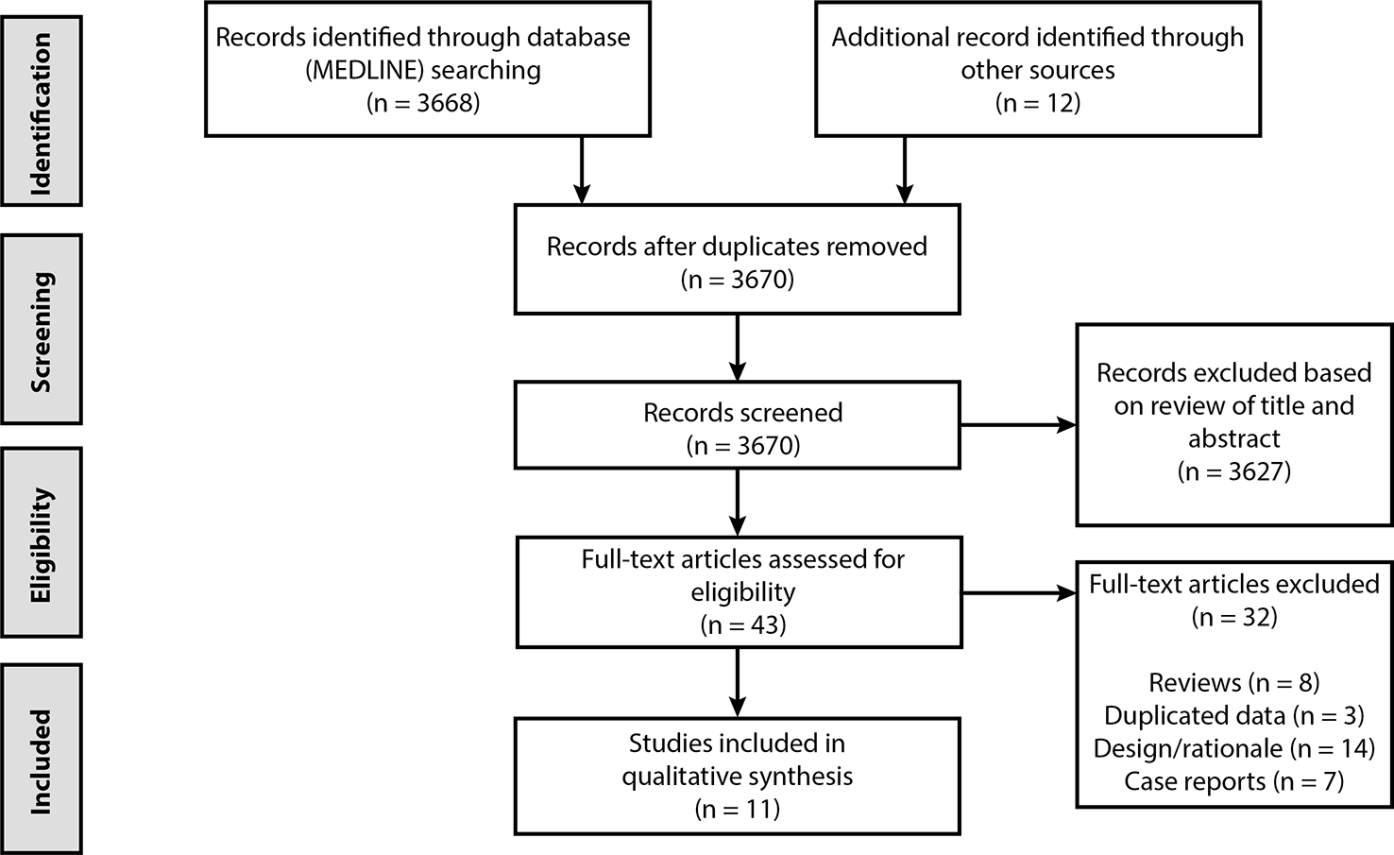
PRISMA flowchart of selected abstracts and articles.

A total of 3,668 papers were identified through the search in Pubmed, and 12 additional records were found through other sources (manual search based on review papers and meta-analysis). After the removal of duplicates and exclusion based on titles or abstracts, 43 papers were screened in more detail for eligibility. Subsequently, another 32 were excluded, which resulted in the 11 papers used in this systematic review. This process is described in the PRISMA flowchart (**Figure 1**). The quality of the great majority of the clinical trials found did not conform to modern standards, with a non-randomized control group or without control group itself. The highest quality of trials was observed in studies on the therapeutic use of LSD in alcoholism.

The detailed description of all studies included and their main results can be found in **Tables 1** and **2**.

**Table 1:** Details of studies: design, diagnosis and measurement.

Clinical Trial; (Country)	LSD dosage (n)	Control (n)	Blinding	Target condition/Inclusion criteria	Measures (time horizon)
Smart et al. (68); (Canada)	800 mcg (10)	60 mg ephedrine sulfate (10) No drug (10)	Double-blind (not to “no drug” group) Independent assessors	Alcoholics, “long history of excessive and uncontrolled drinking” (Male and female)	Drinking History Questionnaire, Abstinence (6 months) Maudsley personality inventory, Haigh-Butler Q, Rorschach, Wechsler Adult Intellingence Scale
Hollister et al. (69); (USA)	600 mcg (36)	60 mg d- amphetamine (36)	Double blind Independent assessors	Alcoholic Veterans, “acute alcoholic episode within 2 weeks of admission; all problem drinkers” (Male)	Drinking Behaviour Scale (2, 6 months)
Ludwig et al. (70); (USA)	3 mcg/kg 210 mcg mean (132)	No drug (44)	Double blind until LSD session Independent assessors	Alcoholics, “up to 4 previous admissions for treatment of alcoholism” (Male)	Behaviour Rating Scale (6, 12 months) Abstinence (1, 3 months) California Psychological Inventory
Johnson (71); (Canada)	300 mcg initial dose + 264 mcg mean (48)	3.75 g Sodium Amytal + 30 mg Methedrine (22) / No drug (25)	Single blind Independent assessors	Alcoholics in outpatient treatment (Male and female)	Abstinence, Drinking practice/consequences (12 months) Differential Personality Inventory, Quick test, Hidden Figures test
Bowen et al. (72); (USA)	500 mcg (22)	25 mcg LSD (22) No drug (15)	Double-blind Independent assessors not mentioned	Alcoholic Veterans under voluntary treatment for alcoholism (Male)	Adjustment scale (12 months)
Denson and Sydiaha (73); (Canada)	50-300 mcg (163 mean) in subsequent dosage + 5 mg dextroamphetamine prior to LSD (25)	No drug (26)	No attempt of blind Independent assessors	Alcoholic and neurotic patients (Male and female)	Eysenck Personality Inventory, IPAT Objective Anxiety Scale, Minnesota Multiphasic Personality Inventory, Lorr Multi-dimensional Rating Scale, Background Questionnaire for Non-Schizophrenic Patients (BFQNSP) (6, 12 months)
Pahnke et al. (62); (USA)	450 mcg (73)	50 mcg LSD (44)	Double-blind Independent assessors	Alcoholics under voluntary treatment for alcoholism (Male)	Drinking Behaviour Scale, Global Adjustment (6 months)
Tomsovic and Edwards (59); (USA)	500 mcg (32) *non-schizophrenics	Usual treatment (45) *non-schizophrenics	Double-blind until LSD sesión Self-report assessment	Alcoholics with 12 years average of problem drinking (Male)	Drinking Adjustment Scale (3, 6, 12 months) Blewett and Chwelos Scales
Savage and McCabe (74); (USA)	300-450 mcg (37)	Usual treatment (37)	No attempt of blind Independent assessors	Narcotic addicts in Maryland correctional institutions (Male)	Global adjustment rating scale, Abstinence (6, 12 months)
Savage et al. (65); (USA)	350 mcg (31)	50 mcg LSD (32) Usual treatment (33)	Double-blind Independent assessors	Patients with psychoneurotic diagnosis, “depressed and anxious” (Male and female)	Psychiatric evaluation profile, Katz Adjustment Scale, Global adjustment scale (6 months) Block Design, Digit Span, Digit Symbol, Progressive Matrices, Embedded Figures, Benton Visual Retention Test, Minnesota Multiphasic Personality Inventory, Eysenck Personality Inventory, Personal Orientation Inventory
Gasser et al. (75); (Switzerland)	200 mcg (8)	20 mcg LSD (3)	Double blind Independent assessors	Anxiety associated with life- threatening deseases patients (Male and female)	State-Trait Anxiety Inventory, European Cancer Quality of Life Questionnaire, SCL-90-R, Hospital Anxiety and Depression Scale, (1 week, 2, 12 months)

**Table 2:** Details of studies: set, setting and main findings.

Clinical Trial; (Country)	Treatment program	Pre-LSD session	LSD session	Setting	Main findings
Smart et al. (68); (Canada)	Therapeutic community (group and individual therapy)	Brief orientation	3h interview and no full-time observation	Waist belt to bed No music/visual stimuli	Improvement in total abstinence/longest period of abstinence for all groups No significant differences between groups
Hollister et al. (69); (USA)	Short therapy on alcohol withdrawal (7 days)	Brief orientation	Brief supportive reassurance (focus on the self)	Music stimuli Visual stimuli (comfortable furniture)	Significant improvement for LSD group (2 months) in Drinking Behavior Scale scores No significant differences at 6 months
Ludwig et al. (70); (USA)	Intensive milieu therapy (30 days) Group therapy	Brief orientation	3h of therapy (psychedelic therapy (44) hypnodelic therapy (44), or silent observation (44))	Not described	Significant improvement in pre-post treatment evaluation for all groups Significant improvement in BRS for all groups in every period No significant differences between groups
Johnson (71); (Canada)	Milieu therapy (24h hospitalization)	Brief orientation	4h of therapy (active interviewing to focus particularly on current problems)	Waist belt to bed No music/visual stimuli	Significant improvement across all groups on most drinking indices No significant differences between groups
Bowen et al. (72); (USA)	Group therapy (60 days)	Group lectures on possible drug effects	Supportive reassurance (focused particularly on non- verbal introspection)	Music stimuli Visual stimuli (flowers, pictures, “tasteful furniture”, mirror)	No significant differences between groups at 1 year after LSD session.
Denson and Sydiaha (73); (Canada)	Not described (24 h)	Not reported	Not described	Not described (general hospital setting)	Positive results in general health (BFQNSP) for LSD group No other significant differences
Pahnke et al. (62); (Canada)	Intensive individual therapy (49 days)	Extensive individual preparation for treatment	Therapy for eliciting a “peak or transcendental experience”	Music stimuli Visual stimuli (flowers, pictures, “comfortable living room”)	Significant improvement in Global Adjustment and Drinking Behavior for LSD group Significant relationship between better Global Adjustment and peak-experiences
Tomsovic and Edwards (59); (Canada)	Group therapy (90 days)	Lectures and reviews of treatment intentions	Supportive reassurance (not focused on extensive talking)	Music stimuli Visual stimuli (flowers, colorful drapes, pictures, hand mirror, scenic view)	Improvement in abstinence for LSD group (significant for control sub-group 1) No differences between lysergide experience measures and benefit
Savage and McCabe (74); (USA)	Brief residential psychedelic psychotherapy (4-6 weeks) in outpatient clinic program	Preparatory psychotherapy (24 h) focused on positive patient-therapist relationship	Psychedelic therapy	Not described	Significant improvement in total abstinence for LSD group Not significant differences in global adjustment scale
Savage et al. (65); (USA)	Brief hospitalization, psychedelic psychotherapy (4-8 weeks)	Preparation based on the psychedelic model of psychotherapy (3-5 weeks)	Psychedelic therapy	Not described	Significant improvement in majority of pre-post-treatment measures for LSD group Not significant differences between groups at 6 months
Gasser et al. (75); (Switzerland)	Continuous psychotherapeutic process lasting several months (outpatient program)	Two preparatory psychotherapy sessions “Set”, based on the psychedelic model of psychotherapy	Psychedelic therapy	Music stimuli Visual stimuli not described “Safe, quiet and pleasant room”	Significant improvement in State-Trait Anxiety Inventory (STAI) scores for LSD group at 2 months Positive trends in reductions in trait anxiety (STAI) at 2 months STAI reductions sustained for 12 months

### Place and Publication Date of the Study

Among the selected clinical trials, 3 were carried out in Canada, 7 in the USA and 1 in Switzerland. **Tables 1** and **2** show these clinical trials ordered by date of publication. Note the important 41-year interval between the study by (63); and the modern study by Gasser et al. (75).

### Quality Assessment of Studies

A summary of risk of bias is presented in **Table 3**. Based on the definitions provided by the Cochrane risk of bias assessment tool (67), no trials were assessed to show a high risk of bias related to sequence generation, and all trials used random assignment. Moreover, all trials attempted to conceal allocation, but most of them were judged to have unclear risk of allocation concealment (63, 65, 69, 71–73) because did not describe methods in detail.

**Table 3:** Quality assessment of all included studies based on the risk of bias.

Clinical trial	Random sequence generation	Allocation concealment	Blinding for participants and personnel	Blinding of outcome assessment	Incomplete outcome data	Selective reporting	Other sources of bias
Smart et al. (68)	Low	Low	Unclear	Low	Low	Low	Low
Hollister et al. (69)	Low	Unclear	Low	Low	Low	High	Low
Ludwig et al. (70)	Low	Unclear	High	Low	Low	High	Low
Hollister et al. (69)	Low	Unclear	High	Low	Unclear	Unclear	High
Bowen et al. (72)	Low	Low	Low	Unclear	High	Low	Low
Johnson (71)	Low	Unclear	High	Unclear	Unclear	High	Unclear
Pahnke et al. (62)	Low	Unclear	Low	Low	High	Low	High
Tomsovic and Edwards (59)	Low	Low	High	Unclear	Low	Low	Low
Savage and McCabe (74)	Low	Low	High	Low	Low	Low	High
Savage et al. (70)	Low	Unclear	Low	Low	Low	Low	High
Gasser et al. (75)	Low	Low	Low	Low	Low	Low	Unclear

Five trials (59, 70, 71, 73, 74) were judged to have a high risk of bias due to blinding of patients or staff. In two of them (59, 70), treatment allocation was concealed only until the time of the possible LSD session, and in the other three trials (71, 73, 74) no attempt of blindness or to single blind was made or designed. The rest of them (62, 65, 68, 69, 72, 75) used double-blind designs with active placebo, but in “Smart et al.” blinding of one of the two control groups (control group without active placebo) was not explicitly described.

All trials were judged to have low or an unclear risk of bias due to independent and blind assessment. In one trial (72) the outcome assessor was not explicitly described as allocation-blind and in another one (59) the assessment was collected by self-report questionnaire, confirmed by telephone interview with a close relative or friend. The rest of them (62, 65, 68–71, 74, 75) had independent and allocation-blind assessors.

Two trials (62, 72) were judged to have a high risk of bias due to incomplete outcome data, because participants were excluded if they did not complete the intended treatment program (72) or if received additional doses of LSD (62).

Four studies (59, 65, 69, 73) had substantial rates of missing participants at follow-up. However, retention rates were generally high, and data missed in one of the trials (63) was only representative at 12 and 18 months, not at 6 months. In the other three trials (59, 69, 73), authors considered missing participants as unimproved.

Three trials (69, 70, 73) were judged to have a high risk of bias because of possible selective outcome reporting, presenting lack of clarity at short-term follow-up clinical outcome and giving more detailed data at medium or late-term follow-up. Another trial (71) was judged to have an unclear risk because some measures were not strictly reported.

Finally, four trials (62, 65, 71, 74) were judged to have a high risk of other sources of bias. In one of them (62), due to baseline imbalance (full-dose LSD participants were less likely to be divorced and more likely to have prior admissions for alcohol treatment), other trial (65) due to treatment time (full-dose LSD participants were more likely to have more psychotherapy hours) and the rest of them (71, 74) due to a shorter time of hospitalization [from one day (71) to a few days (74)] for the LSD treatment group and not the control group. Two last trials (73, 75) presented unclear risk of bias due to uneven concurrent use of other pharmacological treatments during study between participants.

### LSD Dosage and Method

LSD was administered to 567 patients in a dose range from 20 to 800 mcg. The oral route was significantly the most used one, while one study (71) used the intravenous route and another one (68) did not describe the route used. A single dose of LSD was the procedure of choice for most selected clinical trials. Other studies (71, 73) opted for a dosage-escalation approach, and some (73, 75) offered the possibility of repeating LSD doses at 2–3 week intervals.

The concomitant use in some of the studies of other pharmacological principles, such as dextroamphetamine (73) prior to the dose of LSD, or chlorpromazine or promazine (71, 73) after LSD treatment is worth mentioning. Since the therapeutic potential of LSD may be underestimated or masked by such treatments.

### Safety and Adverse Effects

Most studies describe exclusion criteria for patients to be treated with LSD. Severe organic disease (mainly at neurological and cardiovascular levels) was a common exclusion criteria (63, 66–69).

“Gasser et al.” do not rule out those patients with cardiovascular disease, due to the idiosyncrasy of subjects under study (life-threatening diseases). Two of the studies (67, 75) also excluded those patients with a history of severe affective disorder. Most clinical trials (65, 68–71, 74, 75) discarded those patients with active psychosis for the study, but some of them (65, 68, 70, 74) did not rule out patients with a history of psychosis in the past. It is noteworthy that in the study of Tomsovic and Edwards (59), LSD was administered to a subgroup of 12 patients diagnosed with schizophrenia (withdrawn from **Table 1**, due to modern exclusion criteria), to which they applied a separate statistical analysis that showed better results for the subgroup of non- schizophrenics who had received a single LSD dose.

Two cases of serious adverse effects were reported. In one of the studies (69), authors described a tonic–clonic seizure, without subsequent complications, in a patient with a previous history of seizures in a context of abstinent clinical symptoms. In another one (74), a case of prolonged psychosis was reported in a 21-year-old patient with a previous history of recurrent psychotic episodes in the context of hospitalization during adolescence. This patient received psychotherapy and antipsychotic medication, recovering without later complications. No other serious adverse effects were described in the remaining 565 subjects.

### Control Group and Active Placebo

Five studies within our review (68, 70–73) designed a control group for which no drug was administered, and three others (59, 65, 76) had a control group in which the usual treatment was applied to patients during hospitalization. In “Savage et al.” the control group had the added benefit of participating in one hour and a half group therapy sessions three times a week, which were defined as eclectic (focused on the solution of specific problems through group interaction). Most studies (see **Table 1**) had a control group in which active placebo was used, and four of them (62, 65, 72, 75) used LSD itself at a lower dose. The difficulty in maintaining patient and therapist uncertainty, even with active placebo, is underlined by authors. With ephedrine sulfate (68), in 19 of 20 cases the therapist correctly guessed which type of drug was administered to the patient, and 20 mcg of LSD (75) was considered too low a dose to avoid unmasking the control group, both for patient and therapist.

### Treatment Program and “Set”

There was great heterogeneity among the clinical trials chosen for this review in terms of patient preparation and the general therapeutic program to which LSD treatment was added. **Table 2** shows the type of treatment program used in each study, ranging from 24 h to 90 days from the start of treatment to patient discharge. The treatment program between different studies also differed in structure, varying between highly structured intensive programs (70) (with five weekly meetings, seminars, group and individual therapy, occupational therapy and rehabilitation program) and the absence (73) of a specific program.

Preparation of the subject for LSD treatment ranged from very brief orientation (68–71 to extensive preparation (62, 65, 74, 75) with the aim of promoting the therapeutic experience. Preparation time (pre-LSD session, **Table 2**), ranged from a few hours to 5 weeks. The only information provided to subjects in some cases was the great variation in the individual response of the drug (68), or very brief data on the nature of response (69), with no intention to perform previous therapy. One of these authors (70) points out that the previous preparation of patients to LSD administration was possibly insufficient for achieving therapeutic objectives.

Despite heterogeneity, there was a trend among most modern trials within our review to emphasize the importance of the “set” of the subjects to be studied, devoting more time and providing them with a structure. In the earliest study meeting these characteristics (72), patients were previously informed of the nature of the drug, stating whether they would receive a small or a large dose. Within the LSD group of treatment (full-dose or active placebo), approximately half of the patients performed the session during the first 3 weeks, with the remaining subjects receiving LSD treatment during the last 3 weeks. There was a non-significant trend towards better results among those who received treatment during the last 3 weeks, which was highlighted by the authors as a positive association between “set” and therapeutic outcomes.

### Therapeutic Approach and “Setting”

#### Therapeutic Approach

Again, great heterogeneity was observed among studies regarding the therapeutic approach during the treatment with LSD. Two studies (68, 71) applied an approach based on active and directed interviews focused on problems derived from alcohol dependence. In one of these trials (68), these interviews were described as an attempt to discover alternatives to alcohol use, and to define patient attitudes regarding the transfer with the therapist, the act of moving towards drinking, parental relationships, suicidal ideation or sexual behavior.

In three of the studies (59, 69, 72), no psychotherapy attempts were made during the treatment session. In one of them (69), an effort was made to maintain a supportive environment, which included non-verbal communication. In another study (70), three different approaches were used during the LSD session, defined as “psychedelic therapy”, “hypnodelic therapy” and “silent observation”, to study possible differences in their therapeutic potential. The author described “an active, dynamically oriented psychotherapy, with the primary focus on major problem areas”, which contrast with the description of “psychedelic therapy” considered above. The most common approach among these studies (62, 65, 74, 75) was to use psychedelic therapy, defined as 12-14 h after one relatively high LSD dose (200-500 mcg), during which a nurse and a therapist provide constant attention (65) with the aim of the subject achieving a “peak or transcendental experience” (62).

#### Setting

Regarding the physical (sensory stimuli) and interpersonal environment of subjects during the LSD treatment (see **Table 2**), in five trials (59, 62, 69, 72, 75), musical stimulation during the session was offered. Descriptions of environment were varied, finding “comfortable or tastefull furniture” (62, 69, 72) or “flowers and pictures” (59, 62) as examples. In four of the studies (65, 70, 73, 74), the physical environment was not described. Likewise, in two studies (66, 69), the use of waist belt to bed method was mentioned to prevent subjects from leaving their position. Regarding the interpersonal environment, the fact that in the earliest study (68) subjects were unaccompanied for an indefinite period of time during the treatment is noteworthy.

### Efficacy

The efficacy of the intervention with LSD was presented by the main diagnosis where the substance was administered.

#### Alcohol Use Disorder

Most clinical trials in this review (59, 62, 68–73) evaluated the therapeutic potential of LSD in the treatment of alcohol use disorder. The main outcomes of these studies and their main statistical analysis were summarized below, by order of publication.

In the study by “Smart et al.” there was a substantial improvement in abstinence (total abstinence and longest period of abstinence) in all three groups [LSD group (800 mcg), active placebo group (60 mg ephedrine sulfate) and “no drug” group], but no significant differences were found between them (ANOVA, p > 0.05). There were no significant differences between groups either in the Drinking History Questionnaire nor in number of voluntary contacts with the clinic afterwards.

The second study (69) showed a significant improvement (t-test, p < 0.01) in the 2-month follow-up in the LSD group with respect to dextroamphetamine, based on the Drinking Behavior Scale score. No significant differences were found at 6 months follow-up, except for two specific symptoms of this scale (related to work performance), in which LSD was shown to be superior to dextroamphetamine (chi-square, p < 0.05).

Conversely, in the study by “Ludwig et al.”, results showed a significant improvement at two weeks of treatment (t-test for correlated means, p < 0.05) for all four groups (three different approaches in LSD group (Hypnodelic therapy group, Psychedelic therapy group and Silent Observation group) and control group). However, no significant differences were found between them (ANCOVA, no alpha value reported). In the same way, a significant improvement (t-test for correlated means, p < 0.05) was observed in the Behavior Rating Scale values for each period (6, 12 months) in all groups, without finding significant differences (ANCOVA, no alpha value reported) between them.

In the next study (71), a significant improvement was found in terms of abstinence (ANOVA, p < 0.01), drinking behavior (ANOVA, p < 0.01) and employment rate (ANOVA, p < 0.05) after treatment in all groups (LSD treatment group, active placebo (Sodium Amytal and Methedrine) control group, and “no drug” control group). However, no significant differences (chi-square, p > 0.05) were found between them. In the same direction, in the study by “Bowen et al.”, no significant differences were found between groups (chi-square, p > 0.05).

In the study by “Denson et al.”, no significant differences (chi-square, p > 0.05) were observed between groups (LSD group and control group) at follow-up, except in the Background and Follow-up Questionnaire for Non-Schizophrenic Patients (BFQNSP) data, in which the LSD treatment group showed better results in terms of general health (chi-square, p < 0.05).

In the next study (62), significant improvements were observed in Global Adjustment (ANCOVA, p < 0.05) and Drinking Behavior (ANCOVA, p < 0.025) for the LSD treatment group compared to the control group at 6 months.

Finally, in the last trial (59), a higher percentage of abstinence was observed among the LSD treatment group compared to the remaining groups (control group 1: no treatment, only ongoing follow-up evaluation; control group 2: usual treatment, “Regular Alcoholic Rehabilitation Program”) at three months, maintaining this superiority at one year in several grades. A statistical difference (chi-square p < 0.01) was observed between the LSD group and the control group 1, but authors emphasized that the control group 1 was not representative of the best results observed in the control group 2.

In summary, it was observed a significant effect of LSD in four studies performed. However, this effect was related to quality of life and general health in some of the studies, with no clear improvements in alcohol abstinence.

#### Neurotic Symptoms (Anxiety, Depression, and Psychosomatic Diseases)

Two trials (65, 73) evaluated LSD as a treatment of neurotic symptoms. This diagnosis was referred to as depressive neurosis, obsessive-compulsive reaction, phobic reaction, anxiety state, hysteria, psychoneurosis with somatic symptoms, character disorder and sexual neurosis. The presence of all symptomatology was not required, and a subset of neurotic symptoms was adequate. “Denson et al.” found significant differences (chi-square, p < 0.05) in Questionnaire data (BFQNSP), in which the LSD treatment group showed better results in terms of general health at 6 and 12 months. Also, in the study by “Savage et al.”, a significant improvement (chi-square, p < 0.05) was observed at 6-8 weeks in most of measurements used for all three groups (LSD treatment group, active placebo (LSD) control group and “usual treatment” control group). This improvement (mainly focused on symptomatology and self-actualization) was significantly greater as an average for the LSD treatment group compared to the “usual treatment” control group, as well as for some measurements used for the active placebo (LSD) control group compared to the “usual treatment” control group. The LSD treatment group showed superiority (chi-square, p < 0.05) with respect to both control groups in a sub-scale of the Minnesota Multiphasic Personality Inventory (F scale, focused on general psychopathology). Regarding subsequent evaluation (6 months), all groups showed significant differences in a large number of variables, but in this case the results of the statistical analysis failed to reach the defined significance level (ANCOVA, p > 0.05) between the groups.

#### Heroin Use Disorder

Only one study (74) met the inclusion criteria in our review. Significant differences were observed (chi square, p < 0.05) in total abstinence rates in favor of the LSD treatment group at 12 months. A trend, not statistically significant (chi-square, p < 0.02), was observed in favor of the LSD treatment group in Global Adjustment Rating Scale.

#### Anxiety Associated With Life-Threatening Diseases

A modern study (75) assessed anxiety associated with chronic inflammatory disease, chronic motor disease and cancer. All patients had a score of 40 and above in the State-Trait Anxiety Inventory (STAI). A positive tendency in trait anxiety reduction (ANOVA, p = 0.033) in the STAI was observed at two months post ingestion, as well as a significant reduction (ANOVA, p = 0.021) in state anxiety in the STAI. Reduction trends in the STAI were maintained after 12 months in the LSD group, however with no significant difference (ANOVA, p > 0.05).

### Aftercare Related to Experimental (LSD) Sessions

In some studies (69, 73) patients could be discharged after 24 h or in less time (73) if they were able to be assisted by friends or relatives. Other studies did not specify which patients maintained subsequent therapy (70), or did not examine session results unless patients actively requested it (68). In one of these studies (70), a possibly inadequate follow-up of subjects was mentioned, without giving them the opportunity to receive further treatment.

One of the authors (72) suggested that short-term changes that occurred frequently in subjects' personality could be integrated and applied to their daily-life insight with greater support and additional help after hospital discharge. In one study (65), patients remained hospitalized at least one week after the LSD session, being visited by their therapists repeatedly. In this study, a second session with LSD was offered to those patients who were considered suitable for second exposure (approximately 25% out of both LSD groups (full-dose and active placebo) received an equal second dose). In another study (75), a second dose was also offered to subjects in the active placebo group at months of follow-up (open- label cross-over design). Finally, in one of trials (70), half of each group was also treated with disulfiram (daily dose of 500 mg) after hospital discharge. Patients were strongly encouraged to take a fixed, prescribed dosage every day, instructed on the dangers of imbibing alcohol while on disulfiram, and started on the drug four days prior to hospital discharge. They were given a six-month supply of disulfiram and instructed to take one 500 mg tablet per day. Baseline to post-treatment t-tests revealed significant improvement (t-test for correlated means, p < 0.05) in Behavior Rating Scale for every group at every period, while two-way analysis of covariance revealed no significant differences (ANCOVA, no alpha value reported) between groups that received disulfiram and those that did not after hospital discharge, for any of the measurements studied.

### Variables in Therapeutic Response

Some studies (59, 62, 74) described efforts to predict therapeutic outcomes in relation to an acute hallucinogen experience. In one of them (59), it was emphasized that the methodology used did not manage to measure crucial aspects of the experience that foresee subsequent benefits. In two others, a significant link was observed between values in the Global Adjustment Scale (62) and the probability of optimal adjustment in the community (74) in relation to the achievement of a “mystical or peak experience” during the LSD session. One of these authors (62) identified the LSD dose as a better predictor than the type of experience in his study; although he also pointed out that there was a close link between “peak-experiences” and a higher drug dose.

On the other hand, in two studies (59, 74) it was observed that patients who seemed to benefit from the treatment with LSD did so optimally with more probability. A greater likelihood of complete abstinence from alcohol (59) or optimal adjustment in the community (74) was observed after the LSD treatment.

Finally, one of the authors (65) highlighted that male patients showed a clear improvement in Global Adjustment with as full dose (350 mcg) of LSD at six months post ingestion, while in females, a greater improvement was observed with low doses of 50 mcg (ANCOVA, p < 0.1).

## Discussion

Despite design heterogeneity among the clinical trials in this review, some positive results were observed, revealing the therapeutic potential of LSD in the reduction of psychiatric symptomatology. The vast majority of authors described important positive short-term changes in patients, although in some studies (59, 65, 69) an important homogenization was observed between the LSD treatment group and the control group at long-term follow-up. Some previous studies of lower quality (77) also exemplified a clear improvement in short-term adjustment, with a later tendency to balance results with the control group. However, this is in contrast with the results shown by some authors (62, 74, 75), in which therapeutic changes were maintained at 6–12 months after treatment. Moreover, in a follow-up study (78) beneficial changes were found at one year of follow-up for hallucinogen therapy compared with conventional psychotherapy in adolescent behavior disorders. Numerous studies in healthy volunteers have been carried out within the last decade, and some of them have showed positive effects more than a year after a LSD or psilocybin single dose (79, 80).

The results of this review could conclude that alcohol use disorder patients may benefit from LSD treatment. Other studies with a lower quality control group (patients did not receive a treatment comparable to the treatment group) also found significant differences in favor of LSD treatment in alcoholism (60, 81). Likewise, according to a retrospective analysis of studies published in the late 1960s, LSD is a potential therapeutic agent for the treatment of chronic alcoholism (82). A recent meta-analysis (83) of six of the clinical trials chosen for this review showed the superiority of LSD over placebo in the treatment of alcoholism with an odds ratio (OR) of 1.96 (95% confidence interval 1.36–2.84 OR, p = 0.0003). This study found that a LSD single dose was comparable in terms of effectiveness with the daily intake of naltrexone, acamprosate, or disulfiram in alcoholism treatment (84–86). Other studies in our review also found promising results regarding LSD use for the treatment of heroin use disorder, anxiety, depression, psychosomatic illnesses, and anxiety in relation to life-threatening diseases. Regarding the latter, several authors (56, 57) emphasize the difficulty of designing placebo-controlled and double-blind trials, due to ethical reasons and the nature of the psychoactive intervention.

Regarding the disparity between some results in our review, and as noted by Pahnke et al. (62) “it is essential to keep in mind the differences in procedure among the various methods, not only because of different kinds of experiences being facilitated, but also because of conflicting results that can be correlated with the method used”. LSD invariably involves a complex interaction between drug dosage, set and setting. This link is also objectified in different studies, showing the significant relationship between the therapeutic efficacy of hallucinogens and an adequate set, setting and integration of later experience (62, 87–90). This could explain some differences between the results of these reviewed trials, in which there was a great variation between the approach of “Smart et al.”, (Psychedelic–chemotherapy: no attempt of psychotherapy, waist belt) and that of “Savage et al.” (psychedelic therapy: set, setting and aftercare related to the LSD session). Some authors (91) argued that the accepted methods proven to generate some beneficial experience with LSD are far from those used by Smart at the 1960s. Therefore, the inherent difficulty in conducting a double blind controlled clinical trials with LSD should be mentioned. In 1964, Whitaker (92) stated his opposition to the design of a control group with this type of substance, due to the promising responses of first patients as opposed to the control group. Due to this difficulty, widely discussed at the time, many studies previous to that carried out by “Smart et al.”, did not apply adequate measures or assessments, without a control group or properly designed statistical analysis. In this regard, Tomsovic and Edwards (59) mentioned “the complexities and difficulties of achieving control over the placebo effect of a drug that has spectacular mind-altering properties, and where research is contaminated by expectations of benefit”.

Also, modern clinical trials are currently facing a series of problems, which could be summarized as follows (93). Firstly, subjective and objective changes experienced with LSD and the rest of hallucinogens, apparent for both the subject and observer, make performing double-blind tests virtually impossible. Likewise, adequate placebo control becomes extremely difficult due to the absence of such changes in the control group. Strict control of the variables related to the therapeutic benefits of LSD is also necessary. Finally, research with these substances must overcome a series of strict ethical committees and restrictions at the legal level.

When attempting to solve difficulties in terms of blinding and adequate placebo control, a valid approach is an active placebo, using LSD at lower doses (94), an approach already suggested within some of clinical trials in our review (62, 65). This methodology, despite possibly minimizing the effects of LSD when compared to its sole administration, is based on results by numerous researchers who have observed the link between dose and quality and intensity of the hallucinogen response (95–98). Dosage and form of administration, as well as the context in which it is carried out, can be strictly controlled within a hospital setting. The possible effects of microdoses of LSD must be takin into account, possibly limiting its use.

Despite the known unpredictability of hallucinogens, great efforts have been made in recent years to know which variables are associated with the therapeutic value of these substances, finding mystical-type experiences as one of the objectives to be achieved (97, 99, 100). Results of recent investigations show that mystical-type experiences are associated with positive long-term changes after a dose of hallucinogens (33, 79, 99–102). The musical stimuli variable has also been observed as a predictor of mystical-type experiences and positive therapy outcomes (103).

As noted by Gasser (76), designing qualitative studies, not only based on pathology-oriented measurements, is also important to detect variables related to other psychopathological symptoms that can potentially be improved by LSD use (e.g. equanimity, self- assurance and mental strength). Currently, there are validated scales available to measure the quality of the hallucinogen experience, such as the Mystical Experience Questionnaire (MEQ-30) (104) and the Ego-Dissolution Inventory (EDI) (96). The apparently unpredictable nature of these experiences makes studying them in empirical research equally difficult and necessary (14, 104, 105).

Moreover, numerous recent studies with LSD regarding changes in neural networks have been carried out. Modularity and integration networks (as observed in resting- state functional connectivity) have been shown to decrease due to effects of LSD (106, 107). Patterns compared to normal waking consciousness have been demonstrated with LSD (108), and a correlation between subjective reports of “ego dissolution” during LSD and an increment of the overall connectivity and global integration of the brain was found (109). These changes at the cerebral level during the acute effects of hallucinogens have been associated with the aforementioned subjective effects “ego dissolution” and “mystical-type”, and could be related to the wide therapeutic value of these substances (101, 102, 105, 110).

Likewise, multiple modern clinical trials involving other hallucinogens have been carried out in the last decade, mainly with psilocybin. Hopeful results have been found for the treatment of alcohol (111) or tobacco (112) addiction, anxiety in relation to advanced cancer (113) or obsessive-compulsive disorder (114). Moderate doses of psilocybin (200 µg/kg) have been used in some modern studies, either with dose escalation (114) or the same dose in various sessions (113), something reminiscent of the psycholithic therapy used in Europe in the past century. Some possible reasons for the greater use of psilocybin over LSD in modern trials were the shorter duration of one effects of the former (thus avoiding hospitalization) or the greater stigma that prevailed regarding the latter (making it difficult to get economic funds and the approval by ethical committees). Beyond psychiatry, the therapeutic potential of LSD in other medicine fields has recently become evident, as in the treatment of cluster headaches in neurology (115).

As it has been previously pointed out, the homogenization of the therapeutic approach is strictly necessary, and training programs related to research and psychotherapy with hallucinogens have recently been developed (116). Also, there are modern guidelines available for the correct use of hallucinogens in clinical research (31). Therefore, the reborn interest of the therapeutic potential of hallucinogens in modern clinical trials is evident, something proven by the remarkable increase in the number of studies carried out with these substances over the last decade (117).

The present review has limitations. Firstly, only articles written in English were selected; this could imply that articles in other languages were excluded despite the fact that these might have provided valuable information. Furthermore, as mentioned above, most studies were carried out during the past century. Moreover and as previously discussed, there was considerable heterogeneity in their design. Also, differences regarding patient populations, features, and diagnostic methods were noticed. Therefore, due to the lack of studies and the features exhibited by selected research, this review can contribute limited evidence on the topic of interest.

This study comes with its own set of strengths. On the one hand, to our knowledge this is the first systematic review of randomized-controlled trials to assess the therapeutic potential of LSD in psychiatry. On the other, a strict selection of studies was carried out, considering inclusion and exclusion criteria as well as confounding factors. With regards to this and in spite of the heterogeneity mentioned above, the important therapeutic value of LSD is revealed and it is observed to be related to variables controlled by the researcher, such as: set, setting and aftercare related to the LSD session. Another positive aspect of this review is that our results highlight the need for randomized-controlled clinical trials with standardized methods to accurately assess the quality of an acute hallucinogen experience. Finally, this review could serve as a guide for further research involving LSD as a therapeutic agent.

## Conclusions

In conclusion, and despite some controversial results mentioned above, LSD is revealed as a potential therapeutic agent in psychiatry; the evidence to date is strongest for the use of LSD in the treatment of alcoholism. Despite the difficulty of designing double-blind clinical trials with this substance, new studies performed under modern standards are necessary in order to strengthen our knowledge, help erase the stigma that still prevails around these substances and open new doors in the future.

## Author Contributions

JF, FF, ME, MF, and MT designed the review. JF and FF reviewed the abstracts and the papers. JF and ME obtained the data from the selected articles. JF, FF, ME, MF, and MT wrote and reviewed the manuscript.

## Funding

This work was supported in part by grants from Instituto de Salud Carlos III (ISCIII, FIS-FEDER, FIS PI11/01961, FISPI17/01962), ISCIII-Red de Trastornos Adictivos (RTA RD16/0017/0003 and RD16/0017/0010) and The European Commission (HOME/2014/JDRU/AG/DRUG/7082, Predicting Risk of Emerging drugs with in silico and Clinical Toxicology (PREDICT)). Also, Suport Grups de Recerca AGAUR-Gencat (2017 SGR 316 and 2017 SGR 530); Acció instrumental d'Intensificació de Professionals de la Salut - Facultatius especialistes (PERIS: SLT006/17/00014); ME has a Juan de la Cierva research contract awarded by the ISCIII (FJCI-2017-31738).

## Conflict of Interest

The authors declare that the research was conducted in the absence of any commercial or financial relationships that could be construed as a potential conflict of interest.
